# A Primary Telangiectatic Mandibular Osteosarcoma With Germ-Line Malignancy-Associated DNA Damage Repair Gene Polymorphisms: A Case Report

**DOI:** 10.1155/2024/2418888

**Published:** 2024-06-06

**Authors:** Muhammad Tahir, Eric X. Wei, Carlina Madelaire, Alice S. Yu, Guillermo A. Herrera, Rodney E. Shackelford

**Affiliations:** ^1^ Department of Pathology University of South Alabama, 2451 University Hospital Drive, Mobile, Alabama 36617, USA; ^2^ Department of Computer Science College of Computer Science and Engineering University of Central Florida, 4328 Scorpius Street, Orlando, Florida 32816, USA

## Abstract

Primary mandibular telangiectatic osteosarcomas are very rare lesions, with only nine cases reported. Histologically, these lesions show multiple cystic blood-filled cavities traversed by neoplastic bone in septa lined by high-grade malignant cells. Here, we report an 81-year-old woman who presented with a mandibular mass, which was surgically resected and analyzed by histologic examination and whole exome DNA sequencing. A diagnosis of telangiectatic osteosarcoma was given. Comparative sequencing data analysis of paired benign and tumor DNA revealed 1577 variants unique to the tumor DNA, which clustered into several gene families, including those regulating DNA repair and apoptosis. Comparison of benign and tumor DNA revealed many shared gene polymorphisms associated with an increased cancer risk. These included polymorphisms in the ATM, p53, BRCA1, and BRCA2 and many other genes. Interestingly, the patient's family history showed an unusually high cancer incidence, likely related to these cancer risk–associated polymorphisms. To our knowledge, this is the first-time sequencing applied to a mandibular telangiectatic osteosarcoma. Our findings may shed light on the molecular origins of these rare tumors and how they may relate to other tumors in related kindreds.

## 1. Introduction

Osteosarcomas are primary bone tumors composed of malignant mesenchymal osteoblast-like cells which produce a disorganized osteoid and/or bone matrix and whose growth depends on tumor-type specific cancer stem cells [1, 2]. Interestingly, they are the earliest identified human cancers and have been found in a 1.7-million-year-old hominin fossil from South Africa and in 77-million-year-old dinosaur bones [3, 4]. Osteosarcomas are classified based on anatomic location (axial/appendicular, central/surface), histologic grade, and the predominant matrix component; osteoblastic; chondroblastic; or fibroblastic. Uncommon osteosarcoma subtypes include surface osteosarcomas (parosteal and periosteal) and the telangiectatic subtype [1, 2].

Osteosarcomas typically present as firm, tender, growing masses with pain lasting from weeks to months, accompanied by loss of function and signs of hypervascularity, including erythema and venous distention [1, 2, 5]. In most studies, osteosarcomas of different subtypes with the same tumor grade have similar prognoses. Radiographically, osteosarcomas can present as dense sclerotic or radiolucent lesions, often with a mixed “moth-eaten” presentation and calcified areas. About 50% show a “sunburst appearance” due to radiating streaks of osteophytic bone formation [1, 2, 5]. Primary osteosarcomas of the jaw are uncommon, representing 0.8%–2.5% of all osteosarcomas, often presenting at a later age with a lower metastasis incidence and a better prognosis [5, 6]. The telangiectatic osteosarcoma subtype is rare and constitutes 3%–10% of osteosarcomas [1, 5–12]. To our knowledge, only nine primary mandibular telangiectatic osteosarcomas have been described [5, 7–12]. Here, we describe a tenth case of this lesion and its DNA sequencing analysis results.

## 2. Case Report

**Figure 1:**
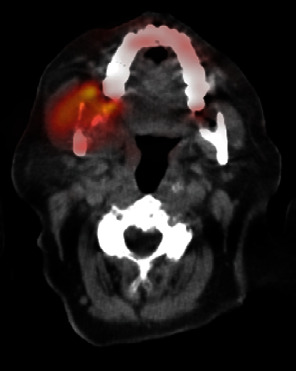
Positron emission tomography/computed tomography with fluorodeoxyglucose avidity in right posterior mandible. A right mandibular exophytic mass measuring 5.0 × 4.0 cm is identified.

**Figure 2:**
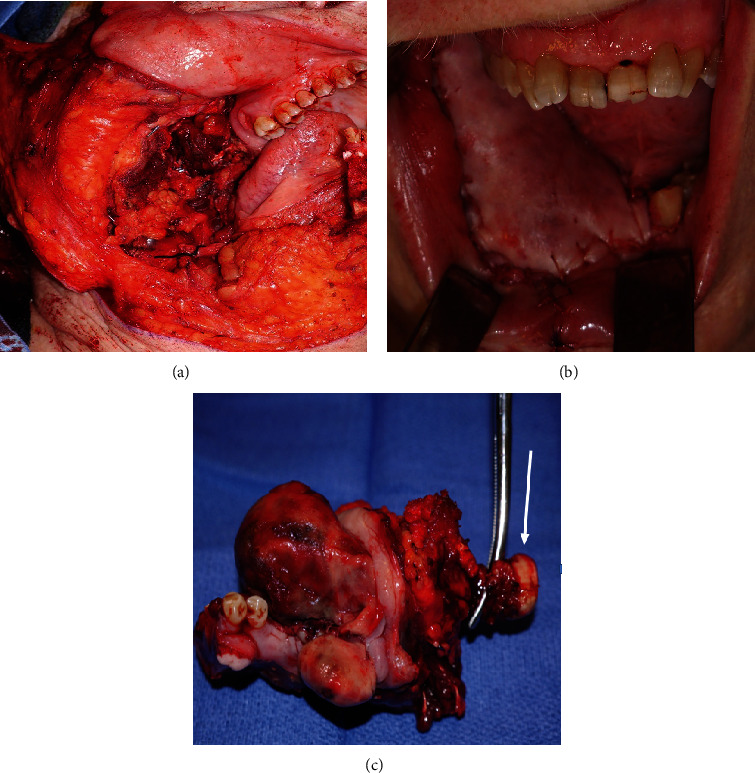
Intraoperative clinical photographs. (a) Surgical defect following composite segmental mandibulectomy with condylar disarticulation via lip split. (b) Surgical defect with mandibular reconstruction plate with alloplastic condylar prosthesis in place. (c) Anterolateral thigh flap in place for soft tissue reconstruction. Photograph of gross specimen (arrow, mandibular condyle).

**Figure 3:**
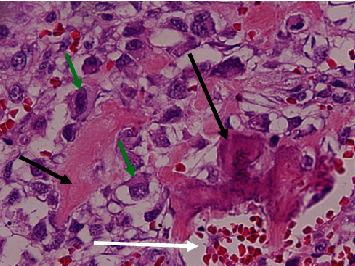
High-power view of hematoxylin and eosin–stained section of the telangiectatic osteosarcoma. The black arrows show osteoid formation. The white arrow shows a blood-filled cyst, typical of this lesion. The tumor stroma is dominated by highly pleomorphic osteoid-producing malignant mesenchymal cells (green arrows).

**Figure 4:**
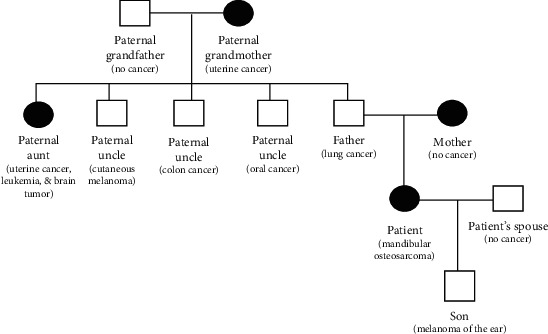
A diagram of the patient's family tree showing an increased cancer incidence descended from the patient's paternal grandmother. Women are depicted with black circles and men with white squares.

An 81-year-old woman presented with temporal region headaches, right mandibular pain, and a slowly growing right mandibular lesion. The patient's past medical history included hypertension, type II diabetes, stroke, and hyperlipidemia. Positron emission tomography/computed tomography imaging revealed an exophytic mass, measuring 5.0 × 4.0 cm (Figure 1). The patient underwent a right segmental mandibulectomy, and the removed specimen measured 10.0 × 6.5 × 6.5 cm and weighed 144 g. The specimen contained soft tissue and skeletal muscle adherent to the mandible, and the mucosal surface was markedly distorted by multiple soft to firm, tan, exophytic nodules, the largest of which measured 3.5 × 3.1 × 2.5 cm (Figure 2). Hematoxylin and eosin–stained sections of the tumor revealed pleomorphic epithelioid cells in a matrix containing osteoid, numerous blood-filled spaces lined by pleomorphic epithelioid cells, with occasional giant cell formation and abnormal mitoses (Figure 3). A diagnosis of moderately differentiated mandibular telangiectatic osteosarcoma with lymphovascular invasion, without lymph node involvement, was rendered, stage pT1, pN0, pathologic stage group IA. Interestingly, the patient's family history revealed a high incidence of cancer in her paternal relatives, including a father with lung cancer; three paternal uncles with colon cancer, oral cancer, or melanoma; a paternal aunt with uterine cancer, leukemia, and a brain tumor; and a paternal grandmother who had uterine cancer. Additionally, the patient's 40-year-old son had melanoma (Figure 4).

Based on the rarity of this tumor and the high cancer incidence in the patient's family, DNA sequencing of the patient's tumor and benign tissue DNA was performed. Tumor and nontumor (benign parotid tissue) DNA samples were collected from the specimen (Figure 2). Tissue was taken for sequencing analysis prior to formalin fixation. The DNA was extracted and examined by Agilent TapeStation 2200. The DNA size distribution ranged from 600 bp to over 45 kb with a peak at 16 kb. The DNA library was prepared using Illumina's Nextera Rapid Capture Expanded Exome Kits. Pair end sequencing was performed at 2 × 76 on Illumina's NextSeq 500 system with Mid Output Kit (150 cycles). FASTQ files were generated by BaseSpace Onsite System. Read alignment and variant call analyses were completed by BWA Enrichment (BaseSpace Onsite System, Illumina) and visualized with Illumina Variant Studio. The quality filter was applied to remove variant calls with low quality. In the tumor DNA, we identified 43,106 single nucleotide variations, 1849 insertions, and 2185 deletions. Seven thousand eight hundred thirty-seven single nucleotide variations were missense variants. Sixty-six deletions and 36 insertions were inframe variants. Comparative analysis between tumor and nontumor DNA disclosed that 1577 predicted deleterious variants were unique to the tumor sample. These variants were seen in coding region, intron, and 3′ or 5′ UTR regions. Among 1577 variants, 801 variants were seen in coding region only, resulting in missense and frame shift mutation, in which 266 variants were homozygous mutations. Some mutants clustered in several gene families, including the mucin (MUC4, MUC6, MUC17, and MUC20), HLA (HLA-A, HLA-B, HLA-C, HLA-DQA, HLA-DQB, and HLA-DBR), zinc finger (ZNF221, ZNF417, ZNF517, ZNF595, ZNF774, and ZNF831), cytochrome p450 (CYP2A7, CYP27C1), DNA repair (GADD45B, MSH4, and TDG), and cell cycle regulating gene families (MCM4, RBBP8NL, PER3, CDK11B, CDC27, CCNE1, and TP53).

In addition to the unique variants in tumor DNA, multiple missense variants in DNA damage/repair-related genes were identified in both the benign and tumor DNAs. These variants may affect DNA repair functions in base excision repair, strand break joining, repair of DNA-topoisomerase crosslinks, mismatch excision repair, nucleotide excision repair, nonhomologous end joining, and homologous recombination. Some of the variants found are listed in Table 1. These shared common variants between benign and tumor DNA indicate that these are constitutional germline variations (polymorphisms) of the patient.

## 3. Discussion

Most osteosarcomas arise de novo, but a subset arises in association with genetic syndromes and other conditions, such as Paget's disease of the bone; Bloom, Werner, Li–Fraumeni, and Rothmund–Thomson syndromes; and chronic osteomyelitis, metallic implants/joint protheses, and high-dose radiation exposures [13]. The telangiectatic osteosarcoma subtype is a rare aggressive, high-grade malignancy accounting for 0.8%–2.5% of osteosarcomas [1, 5]. This osteosarcoma subtype was first described by Paget in 1854, who believed it to be a malignant bone aneurysm. Later, Ewing determined that it is an osteosarcoma subtype [5, 11]. Histologically, they show numerous dilated blood-filled cavities (often over 90%), frequently containing blood clots and/or necrosis, lined by anaplastic tumor cells with osteoid production. The tumor borders are usually well-defined, although invasive growth and soft tissue extension are common [5–12]. There are very few immunohistochemical studies of telangiectatic osteosarcomas; however, one study showed them to be immunoreactive for SATB2 and immunoregulative for CDK4, MDM2, panCK, CD31, desmin, NKX2.2, and TLE1, excluding angiosarcoma, intraosseous rhabdomyosarcoma, Ewing's sarcoma, and synovial sarcoma [10]. To our knowledge, only nine cases of mandibular telangiectatic osteosarcomas have been described (Table 2).

Osteosarcomas show genetic and epigenetic alterations and dysregulation of numerous mRNAs expression, resulting in the disruption of normal osteoblastic differentiation [14, 15]. Kovac et al. [15] analyzed 123 osteosarcomas and identified 14 gene mutations as the main drivers in 87% of osteosarcoma cases, including TP53, RB1, BRCA2, BAP1, RET, MUTYH, ATM, PTEN, WRN, RECQL4, ATRX, FANCA, NUMA1, and MDC1. Interestingly, the osteosarcomas carried large-scale genome instability signatures characteristic of BRCA1/2-deficient tumors, and no one specific mutation was responsible for the majority of osteosarcomas [15]. Only seven molecular studies of telangiectatic osteosarcomas have been performed. Cytogenetic studies have revealed complex chromosomal rearrangements in three tumors and trisomy 3 in one tumor, while metaphase and array comparative genomic hybridizations revealed an average of 2.5 aberrations in two telangiectatic osteosarcomas and regional gains in one tumor at 1q21-12.2, 1q25.2031.1, and 7q21.13-21.1 [16–18]. Here, for the first time, we report DNA sequencing data on this rare tumor.

Sequencing of the tumor DNA revealed mutations clustered in the mucin (MUC4, MUC6, MUC17, and MUC20), HLA (HLA-A, HLA-B, HLA-C, HLA-DQA, HLA-DQB, and HLA-DBR), zinc finger (ZNF221, ZNF417, ZNF517, ZNF595, ZNF774, and ZNF831), cytochrome p450 (CYP2A7, CYP27C1), DNA repair (GADD45B, MSH4, and TDG), and cell cycle regulating gene families (MCM4, RBBP8NL, PER3, CDK11B, CDC27, CCNE1, and TP53). Genes within these families have all been found to be mutated/dysregulated on osteosarcomas, with TP53, MUC4, MUC6, MUC17, TDG, MCM4, PER3, CDC27, and CCNE1 mutation/dysregulation having specifically been previously identified in osteosarcomas [15, 19–26]. Thus, our sequencing results match previous findings and give a detailed molecular analysis of a very rare osteosarcoma subtype. Additionally, the patient's family had an extensive cancer history descended from the patient's paternal grandmother (Figure 4). Comparison of the tumor and benign tissue sequencing revealed many shared gene polymorphisms that conferred an increased cancer risk (Table 1). Thus, the patient's family history of extensive cancer is likely related to these many shared cancer risk–associated gene polymorphisms. Taken together, we present a 10th case of a mandibular telangiectatic osteosarcoma and its sequencing results. We also present data on how cancer risk gene polymorphisms may affect specific individual and associated kindred. Further research will be needed to identify how multiple cancer risk–associated gene polymorphisms give rise to specific tumor types.

## Figures and Tables

**Table 1:** A list of some of the increased cancer risk–associated germline polymorphisms identified in the patient's benign and tumor DNA. The COSMIC (Catalog of Somatic Mutations in Cancer) Program was employed to identify the malignancy types associated with each polymorphism.

**Gene**	**Variants**	**Associated malignancies**	**Gene**	**Variants**	**Associated malignancies**
TP53BP1	D358E K1141Q	GISTs, hematopoietic neoplasms, colon, liver, lung, soft tissue, and pancreatic cancers	RPA1	T351A	Prostatic adenocarcinoma
NEIL3	Q471H G520R	Breast, colon, liver, soft tissue cancers, and GISTs	ERCC2	D312N K751Q	Colon, lung, skin, soft tissue, prostate, and thyroid cancers; GISTs; and hemangioblastomas
APEX1	D148E	Colon, breast, and prostatic cancers and GISTs	ERCC6	R1230P	Gastric and colon cancers; GISTs; and hemangioblastomas
XRCC1	Q399R	GISTs	RAD51D	E253G	Breast, lung, and prostatic adenocarcinomas
DCLRE1A	D317H	GISTs	HUS1B	D268Y	GISTs and gastric adenomas
MLH1	I219V	Colon, prostate, skin small intestine, salivary gland, and soft tissue cancers; lymphoid neoplasms; meningiomas; hemangioblastomas; and osteosarcomas	BRCA2	N372H V2466A I3412V	Breast, bladder, peritoneal, pancreatic, thyroid, colon, gastric, prostate, and soft tissue cancers; mesothelioma; thymomas; meningiomas; rhabdomyosarcomas; GISTs; and hemangioblastomas
MLH3	N826D P844L	GISTs; prostate, lung, and colon cancers; and acute myeloid leukemia	BRIP1	S919P	GISTs, gastric adenomas, and gastric cancer progression
MSH3	Q949R	Colon cancer and tumors of the thyroid and liver	FANCA	T266A	GISTs
MSH4	A97T S914N	Melanoma, colon cancer, and rhabdomyosarcomas	HELQ	V35E	GISTs
PMS1	R202K	Lung adenocarcinomas and hemangioblastomas	MDC1	R268K	Lung cancer
RAD23B	A249V	Osteosarcoma; lung, prostate, and colon cancers; GISTs; hepatocellular carcinoma; and rhabdomyosarcomas	RIF1	V1362M N2021Y	Lung and soft tissue tumors and GISTs
EME1	E69D I350T	Lung, prostate, gastric, and pancreatic cancers; GISTs; and rhabdomyosarcomas	TOPBP1	K457Q N1042S	GISTs and oral SCC
FANCD2	N405S	Lung, thyroid, and colon cancers; acute myeloid leukemia; GISTs; and head and neck SSCs	ATM	F858L P1054R	Mantel cell lymphoma, acute myeloid leukemia, glioblastoma, and prostate, endometrial, Merkel cell, sinonasal, and breast cancers; enchondromas; GISTs; and cervical tumors
BRIP1	S919P	GISTs and gastric cancer progression	BRCA1	E1038G S1634G	Thymic epithelial tumors; breast, prostate, colon, lung, bladder, and penis cancers; meningiomas; GISTs; and acute myeloid leukemia
POLQ	T982R	Colon, prostate, and small intestinal cancers			

**Table 2:** Data on the nine known cases of primary mandibular telangiectatic osteosarcomas in the English literature.

**Study**	**Number of cases**	**Findings**
Naik et al. [5]	1	13-year-old female presented with a swelling jaw for the last 3 weeks. An incisional biopsy showed large and small blood-filled spaces, with neoplastic cells with interspersed osteoid and giant cells. Telangiectatic osteosarcoma was diagnosed, and a hemi-mandibulectomy was performed. The patient was well 7 years later.
Chan et al. [7]	1	15-year-old female presented with a progressive cheek swelling. An aneurysmal bone cyst diagnosed, and the lesion was excised. Four months later, the lesion recurred with extensive soft tissue infiltration by blood-filled cysts. Telangiectatic osteosarcoma was diagnosed. Radiotherapy and Adriamycin were given without clinical effect. The patient died 16 months after presentation.
Tomar et al. [8]	1	9-year-old male presented with a swelling left lower jaw accompanied dull continuous pain for 1 month. On palpation, the swelling was hard and nonmobile. Incisional biopsy revealed blood-filled spaces with calcified osteoid spaces associated with pleomorphic tumor cells. The patient was referred to a higher center for further treatment.
Huvos et al. [9]	1	124 cases of patients with telangiectatic osteosarcoma were collected over a 58-year period. Only one was primary to the mandible (0.8%). No unusual features of this tumor were recorded.
Khan et al. [10]	1	17-year-old female presented with pain and swelling of the left jaw. Examination revealed a 10 × 8 × 4 cm expansile growth, which by CT scan was osteolytic. The lesion contained osteoid with extensive necrosis, numerous large blood vessels, and fibrous septae with abundant atypical cells. The patient was doing well and disease-free 6 months after surgery and chemotherapy.
Ajura and Lau [11]	3	3 of 59 (5.1%) jaw osteosarcomas were primary to the mandible in 14- and 53-year-old males and a 54-year-old female who presented with painless swelling or swelling with paresthesia for the 14-year-old male.
Cheng et al. [12]	1	A 72-year-old woman with a history of Paget's disease of the mandible presented with facial swelling and numbness of the lower lip. A CT scan revealed a lytic lesion. An incisional biopsy revealed pleomorphic mesenchymal cells intermixed with multinucleated giant cells with focal osteoid in a very vascular stoma. Telangiectatic osteosarcoma was diagnosed. The tumor was treated with ahemi-mandibulectomy and chemotherapy. The patient was well 3 years later.

## Data Availability

All data has been included within the manuscript.
